# Antitumor efficacy of triple monoclonal antibody inhibition of epidermal growth factor receptor (EGFR) with MM151 in EGFR-dependent and in cetuximab-resistant human colorectal cancer cells

**DOI:** 10.18632/oncotarget.19797

**Published:** 2017-08-02

**Authors:** Stefania Napolitano, Giulia Martini, Erika Martinelli, Carminia Maria Della Corte, Floriana Morgillo, Valentina Belli, Claudia Cardone, Nunzia Matrone, Fortunato Ciardiello, Teresa Troiani

**Affiliations:** ^1^ Oncologia Medica, Dipartimento di Internistica Clinica e Sperimentale “F. Magrassi”, Università degli Studi della Campania Luigi Vanvitelli, 80131 Naples, Italy

**Keywords:** MM151, cetuximab, acquired resistance, epidermal growth factor receptor, metastatic colorectal cancer

## Abstract

**Purpose:**

We investigated the effect of triple monoclonal antibody inhibition of EGFR to overcome acquired resistance to first generation of anti-EGFR inhibitors.

**Experimental design:**

MM151 is a mixture of three different monoclonal IgG1 antibodies directed toward three different, non-overlapping, epitopes of the EGFR. We performed an *in vivo* study by using human CRC cell lines (SW48, LIM 1215 and CACO2) which are sensitive to EGFR inhibitors, in order to evaluate the activity of MM151 as compared to standard anti-EGFR mAbs, such as cetuximab, as single agent or in a sequential strategy of combination MM151 with irinotecan (induction therapy) followed by MM151 with a selective MEK1/2 inhibitor (MEKi) (maintenance therapy). Furthermore, the ability of MM151 to overcome acquired resistance to cetuximab has been also evaluated in cetuximab-refractory CRC models.

**Results:**

MM151 shown stronger antitumor activity as compared to cetuximab. The maintenance treatment with MM151 plus MEKi resulted the most effective therapeutic modality. In fact, this combination caused an almost complete suppression of tumor growth in SW48, LIM 1215 and CACO2 xenografts model at 30 week. Moreover, in this treatment group, mice with no evidence of tumor were more than double as compared to single agent treated mice. Its superior activity has also been demonstrated, in cetuximab-refractory CRC models.

**Conclusions:**

These results provide experimental evidence that more efficient and complete EGFR blockade may determine better antitumor activity and could contribute to prevent and/or overcome acquired resistance to EGFR inhibitors.

## INTRODUCTION

Epidermal Growth Factor Receptor (EGFR) plays a key role in tumor evolution, proliferation, and immune evasion and is one of the relevant targets for molecularly directed therapy in metastatic colorectal cancer (mCRC) [1–2]. Cetuximab and panitumumab are two monoclonal antibodies (mAbs) that, by targeting the extracellular domain of the EGFR, inhibit ligand binding, receptor dimerization and subsequently activation of downstream intracellular signaling pathways [1, 2]. These two mAbs have been approved for treatment of *RAS* Wild-Type (WT) mCRC [3, 4]. Despite a selection based only upon the absence of any RAS mutations, even in patients who initially respond to EGFR mAbs, progression of disease is inevitable [5]. Various mechanisms which are responsible for the development of acquired resistance in cancer cells have been described, including EGFR gene mutations [6, 7], activation of other Receptors Tyrosine Kinases (RTKs), such as HER2 or MET [8–10], mutation in genes encoding key EGFR-dependent intracellular signaling transducers, such as KRAS, NRAS, BRAF, PIK3CA, MEK or ERK [11–18].

In this respect, the evolution of acquired resistance to anti-EGFR therapy can be defined as the consequence of a perturbation in a system in which most of the mutations that emerge upon treatment involve genes within the EGFR-activated pathways. To escape the perturbation caused by anti-EGFR treatment, cancer cells must settle on a new balance, which is again based on a certain level of EGFR signaling output [2]. These observations prompted the design and development of new approaches including mAb combinations targeting EGFR on multiple, non-overlapping epitopes, that are more efficient than standard anti-EGFR drugs and that are potentially able to overcome acquired resistance [2]. Among these, MM151 is a third-generation EGFR inhibitor consisting of three fully human immunoglobulin G1 antibodies that simultaneously engage distinct, non-overlapping epitopes on EGFR [19]. The use of three antibodies could maximize EGFR inhibition, and may provide mechanisms to overcome resistance to standard EGFR-targeted therapies [20]. MM151 has demonstrated in preclinical models significant EGFR pathway inhibition, as well as enhanced down-regulation of the EGFR [19]. Particularly, MM151 targets regions of the EGFR distinct from those affected by EGFR ECD mutations, which could be a mechanisms of acquired resistance to cetuximab and/or panitumumab [20]. Preliminary phase I results suggest an acceptable safety profile and provide evidence of clinical activity of MM151 in refractory mCRC patients (ClinicalTrials.gov Identifier: NCT01520389).

Based on these considerations, we performed an *in vivo* study by using human CRC cell lines which are sensitive to EGFR inhibitors, in order to evaluate the activity of MM151 as compared to standard anti-EGFR mAbs, such as cetuximab, as single agent or in a sequential strategy of combination MM151 with irinotecan (induction therapy) followed by MM151 with a selective MEK1/2 inhibitor (MEKi) (maintenance therapy). Furthermore, the ability of MM151 to overcome acquired resistance to cetuximab has been also evaluated in CRC models of acquired resistance to cetuximab.

## RESULTS

### Effects of cetuximab and MM151 treatment on human colorectal cancer xenografts

With the aim of developing effective preclinical models for testing possible strategies to prevent and/or overcome acquired resistance to EGFR blockade, we have concentrated our efforts on three human colorectal cancer cell lines (SW48, LIM1215 and CACO2) that are sensitive to EGFR inhibition [15, 16, 21, 22]. In particular, these cell lines function as a relevant model for mCRC patients that would receive cetuximab treatment as none of these cell lines has genetic alterations that are known to be associated with primary resistance to anti-EGFR therapies (*KRAS, BRAF*, and *PIK3CA*) [15, 16, 23–25].

**Figure 1:**
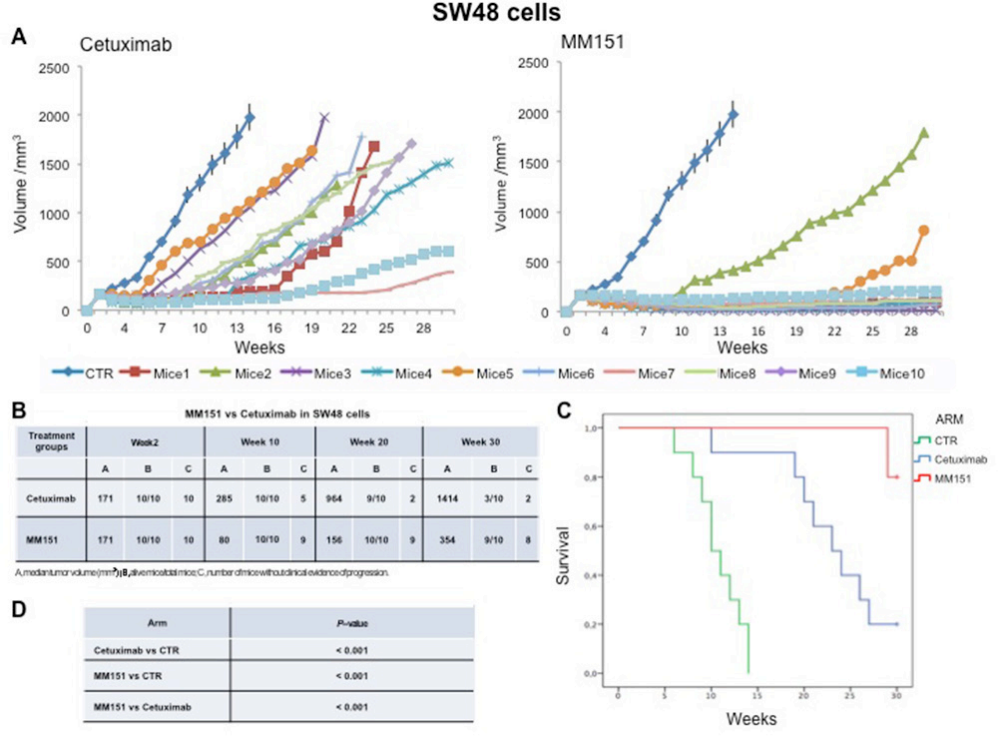
Effects of cetuximab or MM151 on SW48 xenografts **(A-B)** Mice were injected subcutaneously in the right flank with SW48 human colon cancer cells, as described in the Materials and Methods. After two weeks (average tumor size 200-300 mm^3^), mice were treated intraperitoneally with: PBS (phosphate-buffered saline) control, cetuximab, or MM151. The treatment was continued up to 30 weeks after cancer cell injection. Each group consisted of 10 mice. Tumor volumes were measured three times a week. Animals were sacrificed when tumors achieved 2.000 mm^3^ in size. Abbreviations: CTR, control; A, median tumor volume (mm^3^); B, alive mice/total mice; C, number of mice without clinical evidence of progression. **(C-D)** Mice were monitored for survival until 30 weeks following tumor cell injection. Differences in animal survival among groups were evaluated by use of the Mantel Cox logrank test. Cetuximab *versus* CTR, MM151 *versus* CTR, MM151 *versus* cetuximab (*** *p* < 0.05).

**Figure 2:**
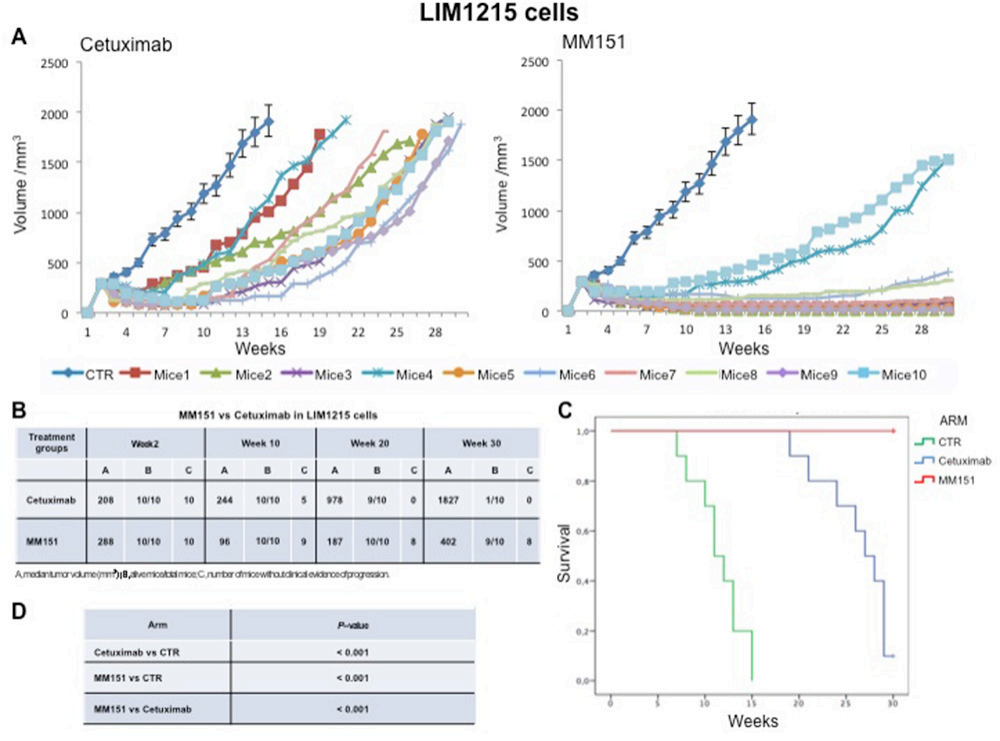
Effects of cetuximab or MM151 on LIM 1215 xenografts **(A-B)** Mice were injected subcutaneously in the right flank with LIM 1215 human colon cancer cells, as described in the Materials and Methods. After two weeks (average tumor size 200-300 mm^3^), mice were treated intraperitoneally with: PBS (phosphate-buffered saline) control, cetuximab, or MM151. The treatment was continued up to 30 weeks after cancer cell injection. Each group consisted of 10 mice. Tumor volumes were measured three times a week. Animals were sacrificed when tumors achieved 2.000 mm^3^ in size. Abbreviations: CTR, control; A, median tumor volume (mm^3^); B, alive mice/total mice; C, number of mice without clinical evidence of progression. **(C-D)** Mice were monitored for survival until 30 weeks following tumor cell injection. Differences in animal survival among groups were evaluated by use of the Mantel Cox logrank test. Cetuximab *versus* CTR, MM151 *versus* CTR, MM151 *versus* cetuximab (*** *p* < 0.05).

**Figure 3:**
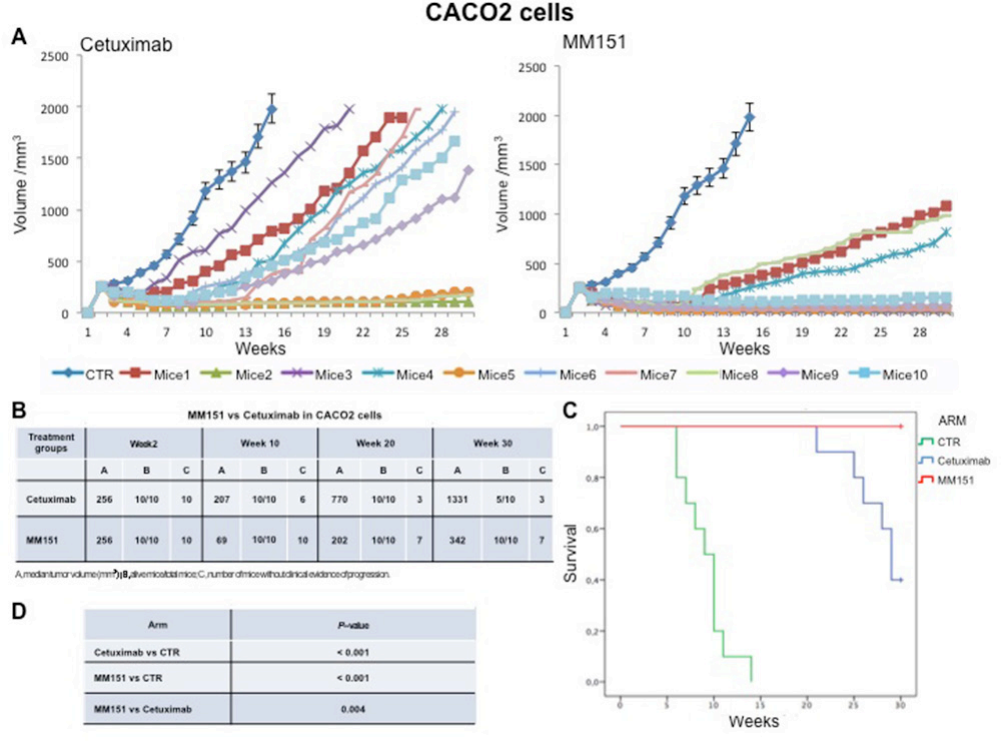
Effects of cetuximab or MM151 on CACO2 xenografts **(A-B)** Mice were injected subcutaneously in the right flank with CACO2 human colon cancer cells, as described in the Materials and Methods. After two weeks (average tumor size 200-300 mm^3^), mice were treated intraperitoneally with: PBS (phosphate-buffered saline) control, cetuximab, or MM151. The treatment was continued up to 30 weeks after cancer cell injection. Each group consisted of 10 mice. Tumor volumes were measured three times a week. Animals were sacrificed when tumors achieved 2.000 mm^3^ in size. Abbreviations: CTR, control; A, median tumor volume (mm^3^); B, alive mice/total mice; C, number of mice without clinical evidence of progression. **(C-D)** Mice were monitored for survival until 30 weeks following tumor cell injection. Differences in animal survival among groups were evaluated by use of the Mantel Cox logrank test. Cetuximab *versus* CTR, MM151 *versus* CTR, MM151 *versus* cetuximab (*** *p* < 0.05).

Cancer cells were injected subcutaneously into the dorsal flank of nude mice. After two weeks, when tumor size was approximately 200-300 mm^3^, 10 mice per group were treated intraperitoneally for 30 weeks with cetuximab or MM151. As shown in Figures 1-3 both agents determined initially in all three models tumor growth inhibition. However, this was more pronounced in the MM151 arm reaching a statistical difference compared both with untreated controls and with cetuximab treatment (FDR corrected p value< 0.05) (Figure 1). The response rate was significantly better in the MM151 group than in the cetuximab group. In particular, in SW48 xenograft, at week 20 the median tumor volume was of 156 mm^3^ in the MM151 group versus 964 mm^3^ in the cetuximab group with 9 out of 10 mice without clinical evidence of progression versus only 2 out of 10 mice, respectively (Figure 1). Similar findings were observed in the LIM1215 and CACO2 xenograft models (Figures 2 and 3).

Further, at week 30, in the MM151 group the median tumor volume was of 354 mm^3^ with 8 out of 10 mice still receiving the assigned treatment in SW48 xenograft model. On the contrary, in the cetuximab group, the median tumor volume was of 1414 mm^3^ with 2 out of 10 mice still in treatment (Figure 1). A similar difference was observed also in LIM1215 and CACO2 xenograft models (Figures 2 and 3). Within the observation time of the experiment, no recurring tumors were detected in any of MM151-treated mice with complete response, demonstrating a long-lasting effect of the treatment. Therapy was well tolerated with no weight loss or other signs of acute or delayed toxicity (data not shown).

Analysis of mice survival was performed. A statistically significant improvement in survival was observed in the MM151 group as compared to control and cetuximab groups (Figures 1-3).

### Effects of MM151 treatment after progression to cetuximab therapy in human colorectal cancer xenografts

**Figure 4:**
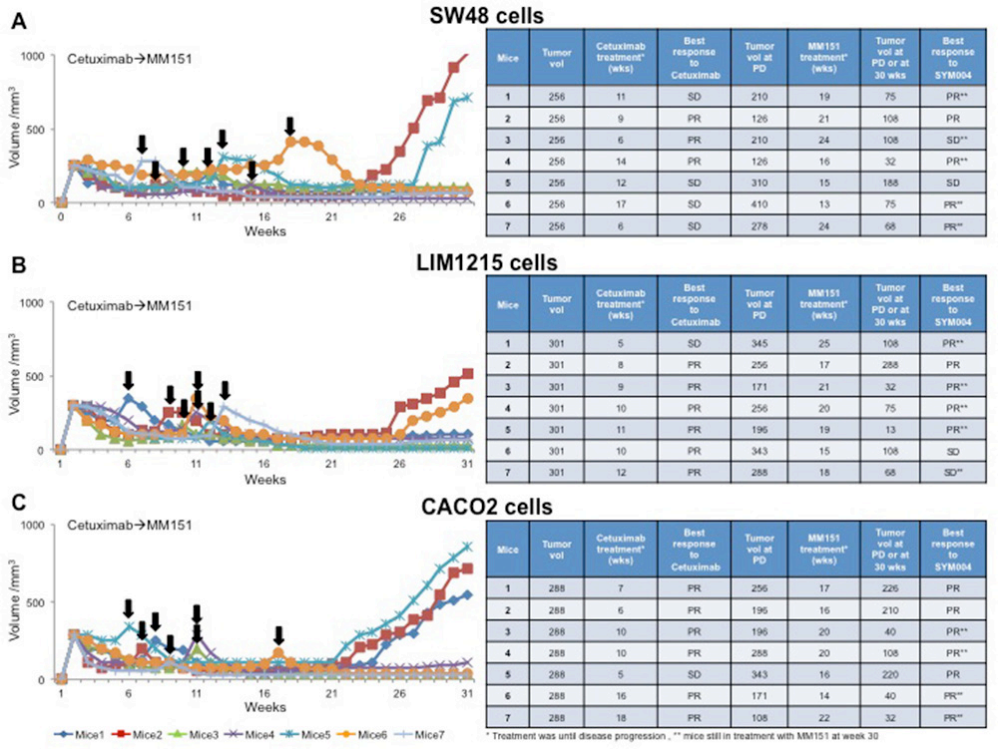
Effect of MM151 treatment after progression to cetuximab therapy in SW48, LIM1215 and CACO2 tumor xenografts **(A-C)** SW48, LIM1215 or CACO2 human colon cancer cells were injected subcutaneously into the right flank of seven nude mice, respectively. After two weeks mice were treated with cetuximab (25 mg/Kg once a week) by i.p. injection. Treatment was continued until disease progression. The black arrows indicate the time of progression to cetuximab. At progression mice were treated with MM151 (25 mg/Kg once a week) by i.p. injection. The treatment was continued until 30 weeks following tumor cell injection. At week 30, five out of seven mice were still on treatment with MM151 in the SW48 and LIM1215 xenograft groups as well as four out of seven in the CACO2 xenograft group (as indicated by double asterisk). Abbreviations: PD, progression disease; PR, partial response; SD, stable disease; wks, weeks.

To test the potential therapeutic activity of MM151 treatment after cetuximab progression, nude mice were injected subcutaneously with SW48, LIM1215 or CACO2 cells. Tumors were allowed to grow to 200-300 mm^3^ and mice were treated with cetuximab until disease progression. At progression, each mouse received MM151 treatment (Figure 4). For monitoring tumor response to therapy, we measured volumetric changes and used an arbitrary classification method partially based on clinical practice, as described in Material and Methods. Progression events occurred in all mice treated with cetuximab. After disease progression, all mice had a benefit from MM151 treatment. In fact, this treatment induced five partial responses (PR) and two stable diseases (SD) in seven mice bearing SW48 or LIM1215 xenografts, respectively, with PR in all seven mice bearing CACO2 xenografts; thus, achieving 100% disease control rate in all three xenograft models. At week 30, 4 out of 10 mice in the SW48 and LIM1215 xenografts and 4 out of 10 mice in the CACO2 xenograft were still on treatment with MM151. No abnormalities were observed in the examined organs, including heart, lung, liver, kidney and spleen derived from all xenograft mouse models (data not shown).

### Antitumor efficacy of irinotecan plus MM151 followed by maintenance treatment in human colorectal cancer xenograft models

**Figure 5:**
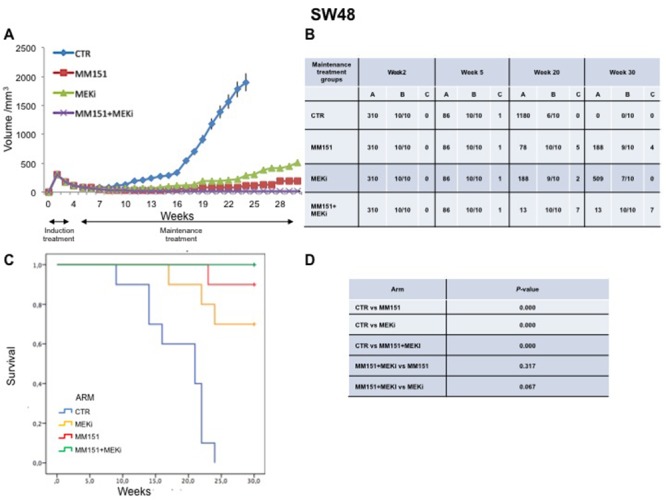
Antitumor efficacy of irinotecan plus MM151 induction therapy followed by maintenance therapy in SW48 xenografts **(A-B)** Mice injected subcutaneously with indicated colon cancer cell lines where treated with irinotecan plus MM151 from week 2 to week 5. Subsequently, from week 5 to week 30 were randomly divided in four groups and treated with indicated drugs. Tumor volume was measured three times per week until week 30 following tumor cell injection. Mice were sacrificed when tumors achieved 2.000 mm^3^ in size. Values are expressed as mean for each group. Abbreviations: CTR, Control, MEKi, MEK inhibitor; A, median tumor volume (mm^3^); B, alive mice/total mice; C, number of mice with clinical complete remission. **(C-D)** Mice were monitored for survival until 30 weeks following tumor cell injection. Differences in animal survival among groups were evaluated by use of the Mantel Cox logrank test. CTR *versus* MM151, CTR *versus* MEKi, CTR *versus* MM151+MEKi, (*** *p* < 0.05); MM151+MEKi *versus* MEKi (** *p* < 0.05); MM151+MEKI *versus* MM151 (not statistically significant).

**Figure 6:**
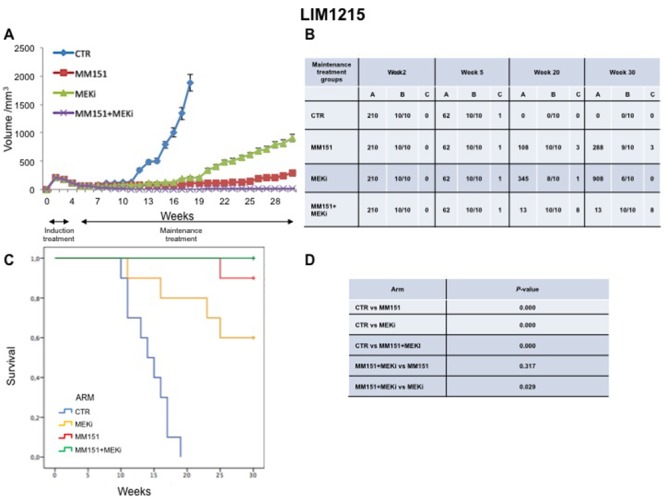
Antitumor efficacy of irinotecan plus MM151 induction therapy followed by maintenance therapy in LIM1215 xenografts **(A-B)** Mice injected subcutaneously with indicated colon cancer cell lines where treated with irinotecan plus MM151 from week 2 to week 5. Subsequently, from week 5 to week 30 were randomly divided in four groups and treated with indicated drugs. Tumor volume was measured three times per week until week 30 following tumor cell injection. Mice were sacrificed when tumors achieved 2.000 mm^3^ in size. Values are expressed as mean for each group. Abbreviations: CTR, Control, MEKi, MEK inhibitor; A, median tumor volume (mm^3^); B, alive mice/total mice; C, number of mice with clinical complete remission. **(C-D)** Mice were monitored for survival until 30 weeks following tumor cell injection. Differences in animal survival among groups were evaluated by use of the Mantel Cox logrank test. CTR *versus* MM151, CTR *versus* MEKi, CTR *versus* MM151+MEKi, (*** *p* < 0.05); MM151+MEKi *versus* MEKi (** *p* < 0.05); MM151+MEKI *versus* MM151 (not statistically significant).

**Figure 7:**
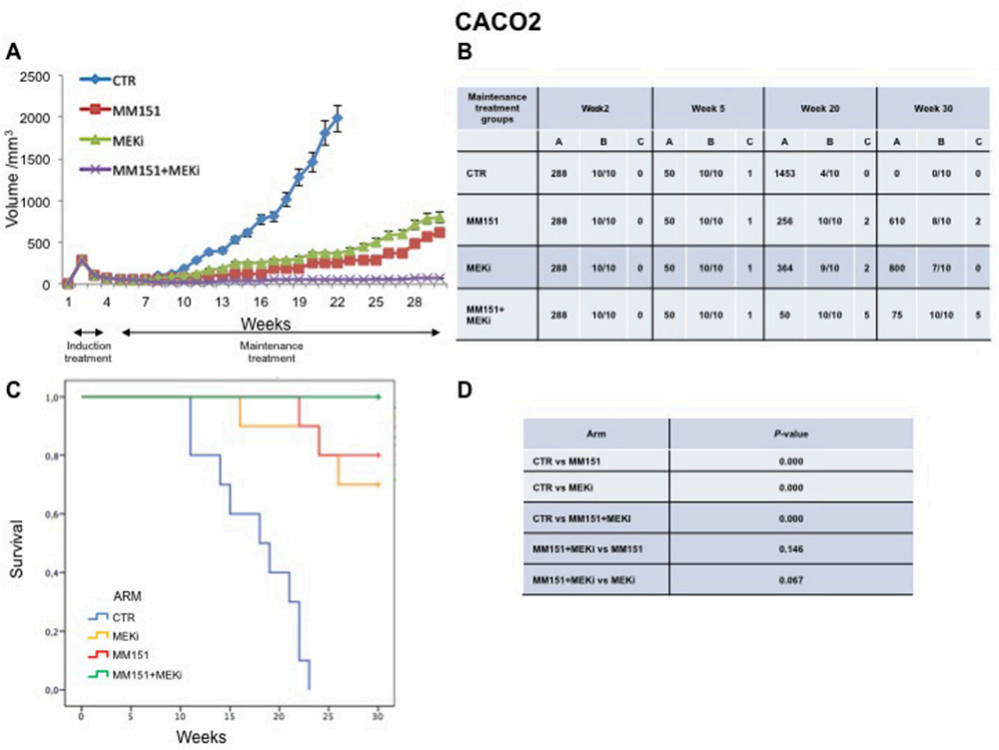
Antitumor efficacy of irinotecan plus MM151 induction therapy followed by maintenance therapy in CACO2 xenografts **(A-B)** Mice injected subcutaneously with indicated colon cancer cell lines where treated with irinotecan plus MM151 from week 2 to week 5. Subsequently, from week 5 to week 30 were randomly divided in four groups and treated with indicated drugs. Tumor volume was measured three times per week until week 30 following tumor cell injection. Mice were sacrificed when tumors achieved 2.000 mm^3^ in size. Values are expressed as mean for each group. Abbreviations: CTR, Control, MEKi, MEK inhibitor; A, median tumor volume (mm^3^); B, alive mice/total mice; C, number of mice with clinical complete remission. **(C-D)** Mice were monitored for survival until 30 weeks following tumor cell injection. Differences in animal survival among groups were evaluated by use of the Mantel Cox logrank test. CTR *versus* MM151, CTR *versus* MEKi, CTR *versus* MM151+MEKi, (*** *p* < 0.05); MM151+MEKi *versus* MEKi (** *p* < 0.05); MM151+MEKI *versus* MM151 (not statistically significant).

In an initial EGFR-sensitive CRC, anti-EGFR treatment can efficiently block the RAS-RAF-MEK kinase pathway [15, 17]. However, acquired resistance is generally caused by the emergence of cancer cells clones with activated mutations in components of the EGFR pathway in order to reactivate this pathway [2, 15, 25]. In this respect, we have previously performed an *in vivo* study using three human colorectal cancer cell lines highly sensitive to EGFR inhibitors, in order to evaluate which maintenance treatment with different inhibitors that act downstream to the EGFR pathway would be able to prevent and/or delay the onset of resistance after an induction treatment with cetuximab plus irinotecan [16]. The combined treatment with cetuximab plus MEKi, after an induction therapy of irinotecan and cetuximab, was able to prevent and/or overcome the resistance to anti-EGFR inhibitors, such cetuximab [16]. Based on the results, we next conducted an *in vivo* experiment in which each of three human cancer cell lines described above was injected subcutaneously into the right flank of groups of 40 female nude mice (Figures 5-7). After two weeks from the injection, mice were treated for three weeks with the combination of irinotecan plus MM151 (induction therapy). Next, mice were randomized in 4 groups (10 mice per group) and treated with vehicle (control), MEKi or MM151 as single agents or with the combination of both drugs, respectively. The maintenance therapy was continued up to 30 weeks from tumor injection in mice. After the three weeks of induction therapy with MM151 and irinotecan all mice achieved a PR. One complete response (CR) was observed in each group of the three tumor xenograft models. Within week 30, all mice treated with only vehicle (control group for maintenance therapy) reached the maximum allowed tumor size of 2000 mm^3^. Among the single agent treatments, the group treated with MM151 showed the greatest tumor growth inhibition in all three tumor xenograft models. In particular, the median tumor volume was of 188 mm^3^, 288 mm^3^, 610 mm^3^, respectively, in SW48, LIM1215 and CACO2 xenografts. The proportion of mice that achieved a CR was greater in the MM151 group than in the MEKi group in all three tumor xenograft models. At the end of treatment, CRs were achieved in 4 out of 10 mice in the MM151 group for the SW48 xenograft, in 3 out of 10 mice for the LIM1215 xenograft and in 2 out of 10 mice for the CACO 2. No CR was registered in the MEKi group in any of the three tumor xenograft models.

As shown in Figure 5-7, maintenance treatment with MM151 plus MEKi resulted in a significant reduction in the risk of progression or death, resulting in the most effective therapeutic modality. In fact, this combination caused an almost complete suppression of tumor growth in SW48, LIM 1215 and CACO2 xenografts with a mean tumor volume of 13 mm^3^, 13 mm^3^ and 75 mm^3^, respectively at 30 week. Moreover, in this treatment group, mice with no evidence of tumor were more than double as compared to single agent treated mice. The delayed tumor growth in the MM151 plus MEKi-treated group was accompanied by a prolonged survival with statistical significant difference as compared either with control or with single agent MEKi groups.

### Effects of cetuximab and MM151 on EGFR-dependent intracellular signaling pathways and on apoptosis process in human colorectal cancer xenograft models

**Figure 8:**
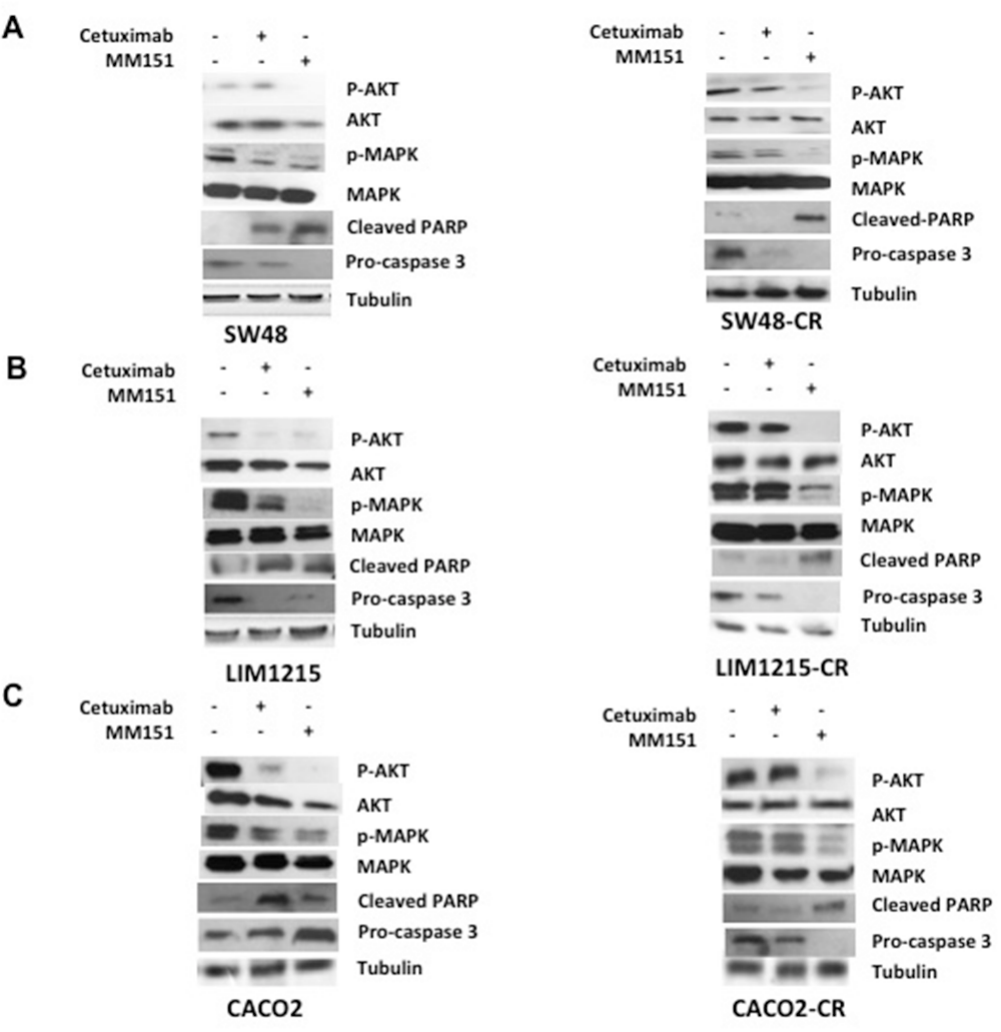
Effects of cetuximab and MM151 on EGFR-dependent intracellular signaling pathways and on apoptosis process in human colorectal cancer xenograft models **(A-C)** Tumors were collected at the beginning of cetuximab treatment and at the onset of resistance to cetuximab from mice engrafted with the SW48, LIM1215 and CACO2 cell lines. As control we used one mouse that has not undergone to any type of treatment from the first *in vivo* experiment. Tumour samples were collected and total cell protein extracts were subjected to immunoblotting with the indicated antibodies, as described in Materials and Methods. Anti-tubulin antibody was used for normalization of protein extract content.

To understand whether the effect of cetuximab and MM151 on EGFR-dependent intracellular signaling pathways, tumors were collected at the beginning of cetuximab treatment and at the onset of resistance to cetuximab from mice engrafted with the SW48, LIM1215 and CACO2 cell lines. As control we used one mouse that has not undergone any type of treatment. First, we assessed the phosphorylation status of EGFR downstream effectors such as MAPK and AKT by Western blot analysis. As shown in Figure 8A-8C, the cetuximab treatment resulted in inhibition of phosphorylated MAPK and AKT proteins only in the cetuximab-sensitive models, whereas no reduction was observed in the cetuximab-resistant models (Figure 8A-8C). On the contrary, the anti-proliferative activity of MM151 was coupled by inhibition of MAPK and AKT phosphorylation in all CRC models tested (Figure 8A-8C). All these findings suggested that MM151 could overcome resistance to anti-EGFR treatment by inhibiting PIK3CA/AKT and MAPK pathways in CRC cancer cells with acquired resistance to cetuximab.

Moreover, to evaluate the pro-apoptotic effect of these drugs, the expression of Cleaved PARP and pro-caspasi 3 have been evaluated. The induction of apoptosis can be followed by a decrease of the pro-caspase 3 and by an increase of Cleaved PARP. The apoptotic effect has been shown with both cetuximab and MM151 treatments in cetuximab sensitive CRC xenografts, on the contrary on CRC cetuximab resistant models only MM151 was able to induce a pro-aptotic effect (Figure 8A-8C).

## DISCUSSION

The importance of EGFR signaling pathway in cancer biology and its potential as a therapeutic target in human cancer is well established [1, 2]. In this respect, although the clinical outcome of *RAS/BRAF* WT mCRC has been improved in recent years for the integration of EGFR agents in the continuum of care, the antitumor efficacy of these drugs is transient and EGFR inhibitor-acquired cancer resistance occurs [2, 5, 25]. There is a clinical need to better understand the mechanisms of resistance to EGFR inhibitors with the aim of defining more tailored treatment modalities to improve survival in patients with mCRC that is initially EGFR-dependent. In this scenario, to overcome acquired cancer resistance to first generation anti-EGFR mAbs, such as cetuximab or panitumumab [5, 25] novel mAbs that could more efficiently block EGFR signaling. MM151 is a third-generation EGFR inhibitor consisting of three fully human immunoglobulin G1 monoclonal antibodies that simultaneously bind distinct and non-overlapping epitopes of EGFR [19].

In a previous experimental study it has been shown that MM151 is potentially more effective than first generation anti-EGFR mAbs in inhibiting EGFR signaling and cancer cell growth, and in inducing EGFR down-regulation [19]. Further, treatment with MM151 can overcome cancer cell acquired resistance to the anti-EGFR mAbs cetuximab or panitumumab, which is sustained by the emergence of EGFR extracellular domain (ECD) mutations [20].

Here we report the results of an *in vivo* study in three RAS WT human colon cancer xenografts as relevant models for EGFR inhibitor-sensitive mCRC to better define the anti-tumor activity of MM151 as compared to cetuximab. For this purpose, we have designed three series of experiments. First, simultaneous triple blockade of the EGFR has a significantly better antitumor activity as compared to the first generation mAb cetuximab. In the second series of experiments, we have found that MM151 had significant therapeutic activity following acquired resistance to cetuximab treatment in all three CRC xenograft models. We have previously demonstrated that an experimental therapeutic strategy consisting of induction treatment with cetuximab plus irinotecan followed by maintenance treatment with cetuximab plus MEKi is effective to significantly delay the onset of acquired cetuximab resistance in the majority of mice bearing human colon cancer xenografts [16]. Therefore, we performed a series of experiments to evaluate the efficacy of this strategy with MM151 in combination with irinotecan and, then, with MEKi. At the ends of this induction treatment with irinotecan plus MM151, mice were randomized to four groups and treated with vehicle, MEKi, MM151 as single agents or in combination. The combined treatment determined an almost complete suppression of tumor growth in SW48, LIM 1215 and CACO2 that lasted up to 30 weeks following cancer cell injection with no evidence of tumors in 8, 7, 5 out of 10 mice, respectively.

These results provide experimental evidence that more efficient and complete EGFR blockade may determine better antitumor activity and could contribute to prevent and/or overcome acquired resistance to EGFR inhibitors [26–28]. Further evaluation of the antitumor activity of the combination of three mAbs that inhibit the EGFR, such as MM151, is of clinical interest in mCRC.

## MATERIALS AND METHODS

### Drugs

Cetuximab, an anti-EGFR human-mouse chimeric monoclonal antibody, was kindly provided by Merck Italy (Rome, Italy). MM151 was obtained from Merrimack Pharmaceuticals (Massachusetts, USA). BAY86-9766 (a selective MEK 1/2 inhibitor) was kindly provided by Bayer Italy (Milan, Italy). BAY86-9766 was dissolved in 0.5% Tween-80 in sterile phosphate buffered saline (PBS) before use. Irinotecan was obtained from the pharmacy of Università degli Studi della Campania L. Vanvitelli (Naples, Italy), dissolved in sterile saline and kept at room temperature.

### Cell lines

The human SW48 (catalogue number: HTL99020) (*KRAS, NRAS, BRAF* and *PIK3CA* wild type) colon cancer cell line was obtained from IRCCS “Azienda Ospedaliera Universitaria San Martino-IST Istituto Nazionale per la Ricerca sul Cancro, Genova” Italy. The human LIM 1215 (*KRAS, NRAS, BRAF* and *PIK3CA* wild type) colon cancer cell line was obtained from Dr. Di Nicolantonio at Candiolo National Cancer Institute (Candiolo, Italy). The human CACO2 (*KRAS, NRAS, BRAF* and *PIK3CA* WT) colon cancer cell line was obtained from Dr. A. Fiorentino at Department of Environmental Biological and Pharmaceutical Sciences and Technologies, Università degli Studi della Campania L. Vanvitelli (Caserta, Italy). SW48 and LIM1215 cells were grown in RPMI-1640 (Lonza) supplemented with 10% FBS, 1% penicillin/streptomycin. CACO2 cell lines were grown in McCoy culture medium (Lonza, Cologne, Germany), supplemented with 10% fetal bovine serum (FBS) (Lonza), 1% penicillin/streptomycin (Lonza). All cell lines were grown in a humidified incubator with 5% of carbon dioxide (CO_2_) and 95% air at 37°C. All cell lines were routinely screened for the presence of mycoplasma (Mycoplasma Detection Kit, Roche Diagnostics, Monza, Italy).

### Tumor xenografts in nude mice

Four- to six-week old female balb/c athymic (nu+/nu+) mice were purchased from Charles River Laboratories (Milan, Italy). The research protocol was approved and mice were maintained in accordance with the institutional guidelines of the Università degli Studi della Campania L. Vanvitelli Animal Care and Use Committee. Animal care was in compliance with Italian (Decree 116/92) and European Community (E.C. L358/1 18/12/86) guidelines on the use and protection of laboratory animals. Mice were acclimatized at the Second University of Naples Medical School Animal Facility for 1 week prior to being injected with cancer cells and then caged in groups of five. We have conduct three different experimental design to evaluate the *in vivo* activity of MM151. In the first one, 3,5 × 10^6^ SW48 or CACO2 cells, or 2 × 10^6^ LIM 1215 cells were suspended in 200 μl of Matrigel (BD Biosciences, Milan, IT): PBS (1:1) and were subcutaneously injected to the right flank of mice. When the mean values of tumors were between 200-300 mm^3^, mice were randomly assigned to one of the following groups (ten mice per group): group 1: vehicle, administrated intraperitoneally (i.p.); group 2: cetuximab, injected once a week i.p. at the dose of 25 mg/Kg; Group 3: MM151, injected once a week i.p. at total dose of 25 mg/Kg. Monitoring of tumor growth was performed until tumors reached approximately 2.000 mm^3^, when mice were euthanized. The treatment was continued for 30 weeks. In the second experiment, groups of 40 mice each were injected subcutaneously with SW48, LIM 1215 or CACO2 colon cancer cells. After two weeks, animals were treated with irinotecan (100 mg/kg once a week, i.p.) plus MM151 (induction therapy). At the end of three weeks of therapy, mice were randomized into 4 groups (n=10 mice per group): group 1: vehicle, administrated i.p.; group 2: MEKi administrated by oral gavage at dose of 25mg/kg, every day for 5 days a week; group 3: MM151, administrated i.p.; group 4: combination of MM151 plus MEKi. This maintenance treatment was continued up to 30 weeks after cancer cell injection. Finally, in the third experiment, three groups of 7 mice each were injected subcutaneously with SW48, LIM1215 or CACO2 cells. Tumors were allowed to grow to 200-300 mm^3^ and mice were treated with cetuximab once a week by i.p. injection. Treatment was continued until disease progression. At progression each mouse was assigned to MM151 treatment. MM151 was administered as above described. The treatment was continued up to 30 weeks after cancer cell injection. In all experiments, mice body weights were monitored daily. Tumor size was evaluated twice a week by calliper measurements using the following formula: π/6 × larger diameter × (smaller diameter)^2^. For assessment of tumor response to treatment, we used volume measurements and adopted a classification methodology loosely inspired by clinical criteria: (i) tumor regression (or shrinkage) was defined as a decrease of at least 50% in the volume of target lesions, taking as reference the baseline tumor volume; (ii) at least a 35% increase in tumor volume identified disease progression; and (iii) responses that were neither sufficient reduction to qualify for shrinkage or sufficient increase to qualify for progression were considered as disease stabilization.

### Immunoblotting

Tumor sample were harvested form euthanized mice, cut into 20-25 mm^3^ pieces and frozen at -80 C in RNA later. Subsequently frozen samples were homogenised in RIPA lyses buffer (0,1% sodium dodecylsulphate (SDS), 0,5% deoxycholate, 1% Nonidet, 100mM NaCl, 10 mM Tris-HCl (pH 7,4)) containing a protease inhibitor cocktail (Hoffmann-La Roche, Basel, Switzerland), 0,5 mM dithiotritol, and 0,5% phenylmethyl sulphonyl fluoride. Tissue lysates were clarified by centrifugation at 14,000 rpm for 10 min a 4 C °. Protein lysates containing comparable amounts of proteins, estimated by a modified Bradford assay (Bio-Rad, Munich, Germany), were subjected to Western blot. Immunocomplexes were detected with the enhanced chemiluminescence kit (Pierce Biotechnology Inc., Rockford, USA). Desired proteins were probed with corresponding antibodies. Cleaved PARP antibody (#9541), p44/42 MAPK polyclonal antibody (#9102), phospho-p44/42MAPK monoclonal antibody (#9106), AKT polyclonal antibody (#9272), phospho-AKT monoclonal antibody (#4060), were from Cell Signaling (Beverly, MA, USA). Anti-pro Caspase 3 antibody (ab32150) was from Abcam (Cambridge, MA, USA). Monoclonal anti-α-tubulin antibody (T8203) was from Sigma Chemical Co. (St. Louis, MO, USA). The following secondary antibodies from Biorad (Hercules, CA, USA) were used: goat anti-rabbit IgG and rabbit anti-mouse IgG. Immunoreactive proteins were visualized by enhanced chemiluminescence. (ECL plus, Thermo Fisher Scientific, Rockford, IL, USA). Each experiment was done in triplicate.

### Statistical analysis

Statistical analysis of *in vivo* data was carried out using the SPSS package (version 21.0 for Windows, SPSS Inc., USA). The Student’s *t* test was used to evaluate the statistical significance of differences between the two treatment group’s effects for each time point considered (2, 10, 20, and 30 week). P values obtained were corrected used FDR method. Survival curves were plotted using the Kaplan-Meier method and compared using the log-rank test. The endpoint was OS. All the tests were two-sided, with P value of <0.05 considered to indicate statistical significance.
